# The Choosing Wisely Initiative and MRIs: Over- and Under-Diagnosis in Japan and Myanmar

**DOI:** 10.7759/cureus.14342

**Published:** 2021-04-07

**Authors:** Takashi Watari, Tin Myo Hlaing, Hideyuki Kanda

**Affiliations:** 1 General Medicine Center, Shimane University Hospital, Shimane, JPN; 2 Healthcare Quality and Safety, Harvard Medical School, Boston, USA; 3 Department of Public Health, Faculty of Medicine University of Miyazaki, Miyazaki, JPN; 4 Department of Public Health, Okayama University Graduate School of Medicine, Okayama, JPN

**Keywords:** physical examination, over-diagnosis, under-diagnosis, mri imaging

## Abstract

Recently, the “Choosing Wisely” initiative has gained traction and high-value care has garnered attention. However, the actual situation of high-value care is different between developed and developing countries. To elucidate this, we highlight the differences between the high-value care provided by healthcare systems in Japan and Myanmar, as representations of developed and developing countries, respectively. Despite the total numbers of magnetic resonance imaging (MRI) equipment in Japan being 400 times higher than in Myanmar, and gross domestic product per capita being 32 times higher in Japan, the individual costs (out of pocket expenditures) per MRI scan are the same (USD 60). However, the total cost per MRI scan is different, implying that differing healthcare costs indicate the existence of over- and under-diagnosis problems among different countries. This study suggests that detailed patient histories and physical examinations are important for selecting relevant diagnostic imaging and reducing unnecessary imaging.

## Introduction and background

High-value care has been promoted by American organizations [1-3]. In 2012, the American Board of Internal Medicine Foundation launched the “Choosing Wisely” initiative to support and enhance conversations between clinicians and patients in choosing necessary evidence-based care and minimize avoidable harm [4-6]. Recently, “Choosing Wisely” has gained traction among medical professionals, and high-value care has attracted the attention of healthcare workers [7]. However, there is limited information on the differences between developed and developing countries with regard to high-value care in clinical situations. Hence, to explain these differences, especially regarding the relevant healthcare systems, we conducted an international comparative and descriptive study through author discussions in Japan (representing developed countries) and Myanmar (representing developing countries). Magnetic resonance imaging (MRI) was chosen as the representative discussion topic. According to Health at a Glance (OECD, 2019) [8], Japan has the largest number of MRI machines worldwide at 55.2 per one million population and is far ahead of the United States (39.1 per one million). Furthermore, access to expensive imaging tests (e.g., MRI) is a clinical feature among developed countries, and modern technological measures, such as computed tomography (CT) and MRI, are rare in medical facilities in developing countries like Myanmar.

Method

For comparing high-value care differences in the healthcare systems of Japan and Myanmar, our study team included a Japanese primary care physician (public health researcher), a primary care physician from Myanmar (public health researcher), and a Japanese public health expert. For the reviews, we used available international data, which were accessed from December 2018 to August 2019 from three electronic databases: MEDLINE (English), Google Scholar (English and Japanese), and ICHUSHI Web (Japanese), including the “explode” function when available and without language restrictions. We used the following keywords to define the inclusion criteria: high-value care, over-diagnosis, under-diagnosis, primary healthcare, Myanmar, Japan, physical examination, diagnostics, and MRI. We collected literature in English, Japanese, and Burmese Myanmar. Two authors independently screened the articles’ titles and abstracts, selecting the ones pertinent to our objectives.

## Review

In 2013, MRI was available to only 0.08 per million of the population, and it was one of the rarest technological devices possessed by countries in the South East Asia region [9]. As resources are limited for high-tech measures, and patients find these services unaffordable, physicians from Myanmar wisely focus on history-taking and physical examination for diagnosis. By contrast, as two-thirds of the hospitals in Japan have high-tech imaging devices available, physicians can easily diagnose patients’ diseases and even provide advanced screening for patients [10]. This widespread availability can lead to cases of over-diagnosis by physicians in Japan and to unnecessary costs for patients as well as governments. However, the main problem arising from excessive scanning is that it can lead to a higher risk of false positives, thereby increasing the danger of unnecessary treatment for patients and increasing the cost burden created by physicians. These consequences suggest a need for more studies like this one to analyze the appropriate use of these devices in Japan.

This study includes a comparative analysis of health resources and uses of MRI in connection to the importance of history-taking and physical examination in Myanmar and Japan. Specifically, to explore the disparity in the numbers of MRI administrations between patients in Myanmar and Japan, this study conducted a comparative analysis of research in health resources, attitudes, and procedures in accessing family medicine using a meta-analysis of existing research in the two countries.

Over-diagnosis: how much access to diagnostic technology does Japan need?

In recent years, as the volume of patients has increased annually with an aging society in Japan, the time physicians spend evaluating patients for diagnosis has decreased, and it is critical for physicians to work in a timely manner. In Japan, with this higher patient volume and the introduction of electronic medical records, physicians may tend to contribute fewer of their skills to diagnosis, especially if diagnostic technology could render the same information. Physicians in Japan are, perhaps excessively, enjoying opportunities to employ high-value diagnostic imaging not only for diagnostic purposes but also for many disease screening processes. Further, patients can freely access health services and treatment in clinics and hospitals without gatekeeping systems like those in the United Kingdom [10]. Having the highest number of high-tech diagnostic imaging scans, physicians in Japan clearly overuse and depend upon these expensive, often unnecessary, tests. For example, a recent study pointed out that about 80% of the radiological testing ordered by physicians needed review for unclear and non-recommended imaging issues, to determine if unnecessary MRI tests could have been avoided, by conducting a more thorough review of the patient’s history [11]. Moreover, with the fear of medical errors or lack of knowledge about the appropriate use of tests, physicians, especially primary healthcare physicians, are routinely supplementing examinations with unnecessary imaging services [12-15].

Such research demonstrates the need to reconsider test ordering processes, particularly the unnecessary imaging that increases national costs in health servicing. The cost, in fact, is a contributing factor that should be considered before imaging can be ordered. Japanese people pay about 30% of such costs as out-of-pocket payments for healthcare services; the other 70% are subsidized by the nationwide, systematic health insurance system and governmental tax [16]. With the annual increase in the cost of high-tech diagnostic imaging, the cost of undergoing an MRI examination should be a point of consideration for both patients and the Japanese government. In 2014, the Japanese national health insurance scheme spent USD 28 million on CT and USD 14 million on MRI examinations [17]. Meanwhile, among developed countries, the cost of MRI is two to three times lower in Japan than in the United States [18].

Under-diagnosis: a great tragedy for Myanmar

**Figure 1:**
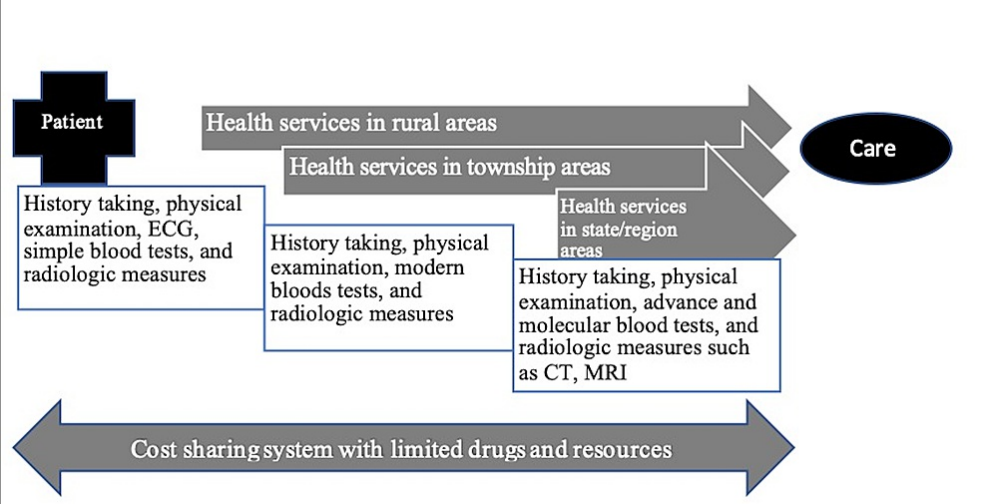
The patient pathway to find healthcare services in Myanmar The nature of accessibility to health care services in rural areas, townships areas, and state/region areas in line with the cost-sharing payment system in Myanmar

People in Myanmar generally rely on basic healthcare services because about 70% of the country’s population lives in rural areas [19]. Moreover, in rural areas, the Myanmar people typically only have access to public health services in clinics that are operated and managed by midwives, as the number of healthcare personnel is insufficient in these areas. To receive services from physicians, a patient must go to a station hospital. Therefore, there is a striking difference between finding healthcare personnel in the rural and urban areas of Myanmar. Moreover, the government subsidizes health insurance only for civil servants, the private health insurance system is not well-organized, and law enforcement by the government of Myanmar is weak. The patient’s pathway to find healthcare services in Myanmar is illustrated in Figure 1, which shows that high-tech diagnostic imaging is only available in big cities.

In Myanmar, not only are human resources for health services insufficient but also resources for infrastructure and facilities [20]. With the annual increase in the volume of outpatient and inpatient care, physicians in Myanmar expend less effort on patient diagnosis due to decreased time for each patient and insufficient access to and feasibility of using high-technology diagnostic tools. For instance, to receive routine imaging, such as an X-ray, patients must go to a hospital, usually located in an urban area. Therefore, it is evident that Myanmar people have difficulties accessing high-tech radiology imaging, especially in rural areas.

Moreover, due to the higher initial price of a new MRI machine, with high installation and maintenance costs and high charges per scan, practitioners in Myanmar can only work with previously used devices from developed countries. This increases the inequality in healthcare services, especially in the rural areas of developing countries and for those who are forced to commute from rural areas to urban areas for obtaining these services. In addition, the lack of professional, high-tech radiologic technicians for MRIs is a major threat to healthcare in developing countries. The high cost of owning and administering MRIs can also lead to unequal distribution among countries. Additionally, the cost of taking MRIs is significantly different, not only among countries but also in private and public hospitals.

Reducing imaging: the importance of patient history and physical examination

With the advent of advanced technological equipment and rising healthcare costs, the art of choosing diagnostic testing wisely has become an essential skill for physicians. However, some physicians are placing less emphasis on history-taking and physical examination in many technologically equipped countries because of a high workload and the similarity of diagnostic information from comparable cases [21]. Importantly, research suggests that physicians must be skilled in bedside exams to make better use of diagnostic tests and avoid unnecessary tests. Choosing wisely among technologies and techniques actually facilitates patient satisfaction and discourages the overuse of unnecessary procedures that are not beneficial to patients and could potentially cause harm from unpredicted false-positive findings [21-23].

With the advancement of imaging techniques (CT, MRI), the use of diagnostic testing approaches - history-taking and physical examinations - to establish the treatment rationale has reduced. However, excessive reliance on routine imaging can affect patient diagnosis and cause unnecessary harm, suggesting the need to incorporate diagnostic testing strategies in treatment [24-29]. Recent studies by researchers have pointed out that about 70%­ to 90% of medical diagnoses of particular cases can be made based on history alone [30], and they have shown that physical exams doubled the diagnostic power of history-taking by 19.5% to ­39%, with an additional increase in diagnostic accuracy of 33% [31]. However, the ordering of too many tests by physicians has been shown to increase stress for patients due to large medical bills. Therefore, in the best interest of the patients, physicians, in general, need to evaluate wisely whether ordering high-cost imaging is necessary by fully utilizing cost-effective diagnostic strategies such as history-taking and physical examination.

In a value-based system where physicians are compensated based on patient outcomes, the only way to manage healthcare-related costs effectively is to improve patient outcomes [32]. Nevertheless, the value necessitates that the cost is affordable for every type of patient. High-cost healthcare measures, such as MRI, are sometimes necessary to elicit conditions and demarcate the region of interest related to the patient’s condition such as detection of aneurysms in brain vessels, breast cancer screening, injuries and abnormalities of the joints, and so on. However, in developing countries and rural areas of developed countries, such measures are rarely available. Therefore, it is necessary for healthcare providers to conduct more of an integrative approach to managing diagnosis by incorporating history-taking and physical examination, even though these practices have their own limitations [33]. Modernized, high-tech diagnostic imaging in healthcare services is noteworthy in terms of achieving an accurate diagnosis. However, the accessibility, feasibility, and costs associated with the use of these devices are indeed exceedingly difficult to manage.

MRI and health resources in Myanmar

Although the area of Myanmar is about 1.8 times [20] larger than that of Japan, the population in Myanmar is less than half of Japan’s population. Myanmar’s healthcare system has been dramatically intertwined with recent reforms in its political situation while Japan’s healthcare system is already well-organized with its nation-wide, systematic health insurance scheme since 1961 [26]. Both nations’ Ministries of Health are the primarily responsible entities for controlling and contributing to the healthcare system. Comparative studies between Myanmar and Japan in terms of healthcare resources and MRI are summarized in Table 1 and Table 2 [10,16,18,20,34-35].

**Table 1:** Comparative studies on healthcare resources between Myanmar and Japan The difference in healthcare facilities and manpower determined the difference in the health status in Myanmar and Japan. These data were extracted from the national-level data of both countries and the current practices of the healthcare sector in 2016.

Total Number of Hospitals	8,842	1,302
Total Number of Clinics (general and dental)	170,469	4,687
Total Number of Patients (outpatients)	496,206,937	10,190,000
Total Number of Patients (in-patients)	457,781,586	2,754,000
Total Number of Doctors	311,205	32,861
Total Number of Nurses	1,149,397	32,609
Total Number of Clinical Radiologic Technologists	44,375	N/A
Doctors per 1000 Population Ratio	2.35	0.6
Nurses per 1000 Population Ratio	9.06	0.53
Clinical Radiologic Technologists Ratio	0.34	N/A

**Table 2:** Comparative studies on the resources and usage of MRI The current practice and usage of MRI in Myanmar and Japan. There is no MRI machine in the rural areas of both countries.

Comparative Studies	Japan 2016	Myanmar 2016
GDP per Capita	$38,972	$1,195
Number of MRIs	6508	16
Number of MRI Exams per Month	961.5	N/A
Total Cost of MRI Exams per Scan	$200	$60/$150*
Cost of MRI per Scan (individual cost)	$60	$60/$150*
Cost of MRI per Portion (public cost)	$140	N/A
Waiting Time for MRI Exams	N/A	2-3 weeks
MRI Accessible Hospitals	300 bedded and more	500 bedded and more

As Table 1 indicates, almost all of the clinics in Myanmar are privately owned, and there are no public clinics for primary healthcare physicians. Therefore, for public healthcare services, patients must seek treatment in public hospitals with primary healthcare physicians. Further, data reflecting the number of clinical radiologic technologists are not available for Myanmar. However, approximately 50 students are selected to train as medical radiologic technicians annually from two medical technology universities of Myanmar (Yangon and Mandalay).

Moreover, Table 2 shows that limited data are available regarding the number of monthly MRI administrations in Myanmar hospitals. Still, this research suggests that MRIs in Japan are administered about 400 times more widely than in Myanmar, and the cost of taking MRIs is different. Nevertheless, the actual out-of-pocket expenditures for MRIs in Japan and Myanmar are the same (USD 60). Comparing the GDP per capita of the two countries, Japanese people earn about 32 times more than the people of Myanmar. This point suggests that MRI imaging is highly costly for Myanmar nationals. For example, one administration of MRI costs nearly 30% of the average salary of doctors in Myanmar. The public cost that is incurred by the insurance scheme for MRI in Japan is about USD 140 per scan, and the increase in scanning also leads to increases in hospital budgets for the government. The cost of taking MRI in Myanmar is not high (about USD 60 in public hospitals and USD 150 in private hospitals), but the maintenance costs for these devices are staggeringly high because few proficient technicians can handle and manage these devices. Therefore, there is a need to hire proficient technicians from foreign countries to maintain these devices. In addition, most MRIs in Myanmar are previously used items from Germany and Japan, not only due to limited government budgets but also because, previously, there were sanctions preventing Myanmar from purchasing high-tech equipment from developed countries, including the United States.

The costs of taking MRI in Myanmar public hospitals have been incurred primarily by private organizations. However, patients can request cost exemptions from the hospitals’ medical superintendents if they cannot afford these costs. Additionally, there are many obstacles to using MRIs in medical check-ups in Myanmar public hospitals such as patients waiting at least two to three weeks. Moreover, the cost of the technology is two to three times higher in private hospitals, and MRI is only available at tertiary hospitals (500 beds), which are primarily located in large cities.

MRI and health resources in Japan

With the excessive number of MRIs and the annual increase in scanning patients, it is clear that physicians in Japan are supplementing their diagnostic testing through high-cost healthcare. Unnecessary use of MRI scanning creates an over-diagnosis problem among physicians, which, in turn, promotes overwhelming workloads for physicians and radiologic technicians and even increases the cost burden for patients and the government. Therefore, the art of choosing wisely in Japan depends on physicians, not only because the scanning instructions are usually given by physicians in both inpatient and outpatient services but also because of the importance of patient satisfaction concerning diseases. In addition, studies show that the overuse of non-recommended imaging is a major challenge in developed countries [36-38]. In fact, the lack of volume restrictions on MRIs may induce unnecessary imaging and increase imaging costs [39].

This study suggests the potential for a golden opportunity to improve high-value healthcare in Japan through greater recognition and understanding among physicians that high-cost imaging alone cannot achieve the most complete patient diagnosis. Since the primary point of consideration for patient diagnosis is history-taking and physical examination, this study suggests that physicians should be advised to focus particularly on primary examinations. With accurate history-taking and physical examination, both physicians and patients can reduce the stress related to radiologic examinations, and the workload for physicians and radiologic technicians can be reduced. Further, patients and physicians can bypass high-valued radiologic examinations and decrease government spending on hospitals. Therefore, this research [30] suggests that physicians who elicit a complete patient history through open-ended questioning and active listening will ultimately obtain critical clues for diagnosis and save many patients from unnecessary imaging.

Discussion

The results of this analysis lead to two important, separate recommended views: one for health policymakers in Myanmar and the other for physicians in Japan. Myanmar policymakers should expand the budget for human resources development and update radiologic measures to overcome severe under-diagnosis problems, especially in rural areas. In contrast, Japanese physicians need to improve their focus on qualitative skills, such as history-taking and physical examination, to eliminate unnecessary radiologic testing of patients [38-39]. However, this descriptive study has some strong limitations. For instance, the Japanese healthcare system has employed a fee-for-service system; thus, once medical institutions have purchased CTs/MRIs, they need to use these devices aggressively to recoup their investment. Moreover, history-taking and physical examination do not generate a profit for clinics/hospitals under the Japanese healthcare system. Furthermore, compared to Japan, Myanmar’s healthcare system, underdeveloped infrastructure, and limited medical research present significant challenges. In other words, in Myanmar, doctors have to diagnose and treat without resorting to expensive tests or existing medical literature, which is not the case in the medical systems of advanced countries like Japan. However, Japan is aging, and medical expenses are increasing. Hence, we believe that high-value care in its true sense is important: “health care is only what is really needed when intervention is needed.”

## Conclusions

This paper analyzed current research on high-cost medical imaging in Japan and Myanmar to better understand the factors that lead to under- and over-diagnosis. Existing research on resources for health, especially MRI imaging, highlights the importance of history-taking and physical examination in solving the problem of over- and/or under-diagnosis and has implications on the situation in Japan and Myanmar. With regard to the use of MRI, this study only analyzed the importance of history-taking and physical examination. Further studies should include analyses on the cost-effectiveness of medical imaging and examine the overuse and underuse of radiologic equipment. While both Japan and Myanmar must choose diagnostic testing wisely, thorough history-taking and physical examination could potentially control unnecessary imaging and provide patient care in underserved regions.
